# Moving towards a uniform diagnosis of coronary artery disease on coronary CTA: Coronary Artery Disease—Reporting and Data System 2.0

**DOI:** 10.1007/s12471-024-01903-6

**Published:** 2024-10-10

**Authors:** Csilla Celeng, Richard A. P. Takx

**Affiliations:** 1 Department of Radiology and Nuclear Medicine, University Medical Center Utrecht, Utrecht, The Netherlands; 2 Department of Radiology and Nuclear Medicine, Amsterdam University Medical Center, Amsterdam, The Netherlands

**Keywords:** Atherosclerosis, Coronary artery disease, Multidetector computed tomography, Risk management

## Abstract

The Coronary Artery Disease—Reporting and Data System (CAD-RADS) is a standardised reporting method which was created in order to improve communication with referring physicians as well as for management considerations. The CAD-RADS score denotes the absence or presence of stenosis, while plaque burden and potential modifiers provide insight into plaque extent and characteristics. The modifier ischaemia enables the incorporation of fractional flow reserve CT and CT perfusion, while the modifier exception is used to denote potential coronary abnormalities. Higher CAD-RADS categories demonstrate incremental prognostic value, with further improvement when taking plaque burden into account. CAD-RADS improves communication with the referring clinician as well as guiding therapeutic management and as such is relevant to uniform patient care in the Netherlands.

## Introduction

According to the 2019 European Society of Cardiology (ESC) guidelines, non-invasive imaging is recommended for all patients with stable chest pain where obstructive coronary artery disease cannot be excluded based on clinical assessment alone [1]. As an initial non-invasive test coronary computed tomography angiography (CCTA) was given a class I recommendation, which resulted in growing demand for high-quality image acquisition as well as reporting standards. Novel guidelines cover a wider range of pre-test probability potentially eligible for referral to CCTA (previously 15–50%). CCTA is recommended from 15% to 30–40% pre-test probability with an option for functional imaging from 30% by a multidisciplinary group of the Netherlands Society of Cardiology (NVVC) and the Dutch Society of Radiology (NVVR) [2]. Although an agreement was reached regarding implementation, there are still substantial discrepancies regarding the interpretation, reporting and communication of results between Dutch hospitals [3].

In recent years, the Coronary Artery Disease—Reporting and Data System (CAD-RADS) was developed in order to standardise reporting of CCTA, to enhance communication between physicians as well as to improve adherence to guideline-recommended therapy. CAD-RADS is an expert consensus document of the Society of Cardiovascular Computed Tomography (SCCT), the American College of Cardiology (ACC) and Radiology (ACR) and the North American Society of Cardiovascular Imaging (NASCI) [4]. Since its introduction it has also been increasingly applied in Europe including the Netherlands; however, its widespread adoption has yet to be established [3, 5]. CAD-RADS quantifies the degree of stenosis and describes the presence of high-risk plaque (HRP) features, stents or bypass grafts. The original CAD-RADS consensus statement was published in 2016 [6]. In 2022 an updated version (CAD-RADS 2.0) was published, which incorporates additional features of atherosclerosis such as plaque burden, the presence of ischaemia as well as results from recent clinical trials [7]. Moreover, the term vulnerable plaque was replaced by HRP features and exceptions were added as modifiers (mainly including coronary artery abnormalities).

**Fig. 1:**
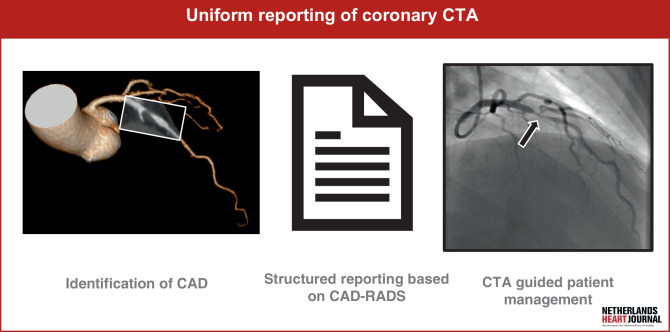
Infographic: Uniform reporting of coronary CT

The following article provides a brief overview on the main aspects of CAD-RADS (Fig. 1), discusses current reporting practices in the Netherlands as well as assessing the potential benefit of using standardised reporting for the development of artificial intelligence.

## CAD-RADS 2.0

**Fig. 2:**
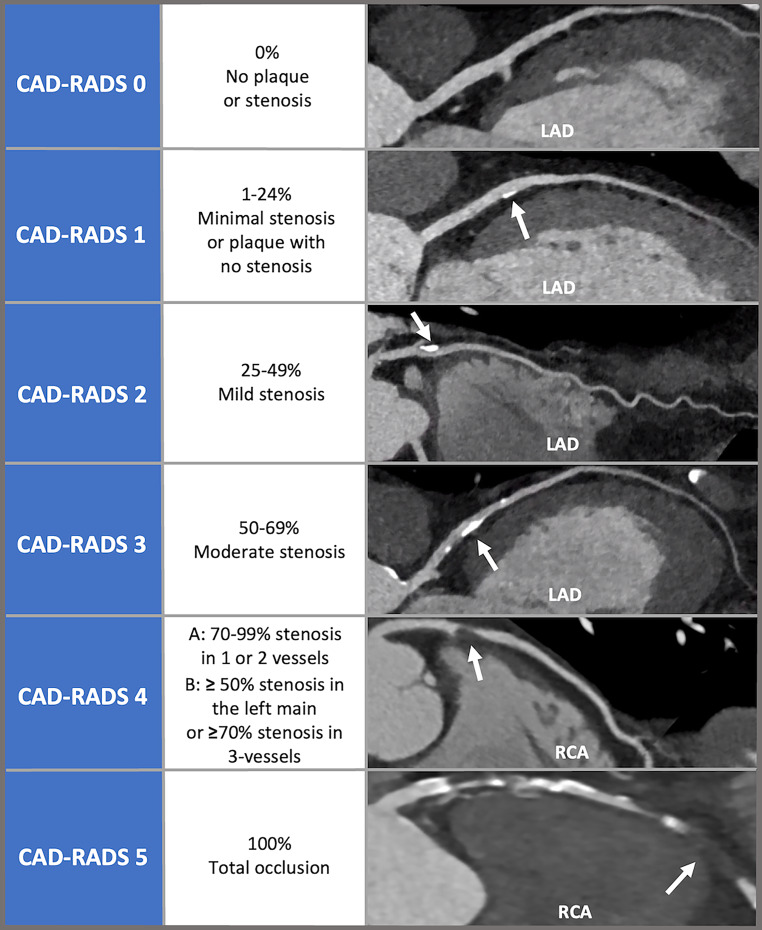
CAD-RADS categories: CAD-RADS stenosis degree with CCTA examples. *LAD* left anterior descending, *RCA* right coronary artery

The main CAD-RADS category is based on the degree of stenosis in vessels with a diameter larger than 1.5 mm. The coronary segments are based on the SCCT segmentation diagram [8]. The different CAD-RADS categories are displayed in Fig. 2. Positive remodelling due to plaque without the presence of stenosis is graded as CAD-RADS 1.

Three types of plaque are categorised on CCTA: calcified, partially calcified and non-calcified (avoid terminology such as ‘soft plaque’). In CAD-RADS 2.0, plaque burden (P) has been added ranging from P1 (mild) to P4 (extensive) (Tab. 1). CAD-RADS 0 denotes the absence of plaque or stenosis, therefore P0 as such is not required. Plaque burden can be determined using calcium scoring, segment involvement score or by visual assessment. The calcium score, however, should not be used as an isolated marker of plaque burden but rather be incorporated in the total plaque burden, especially in case of presence of non-calcified plaques. The preferred method is determined at the local institution. Plaque burden should be listed after stenosis degree (e.g. CAD-RADS 2/P2).

**Table 1:** Coronary plaque burden (P)

	Amount of plaque	SIS	Agatston score	Visual
P1	Mild	≤ 2	1–100	1–2 vessels with mild amount of plaque
P2	Moderate	3–4	101–300	1–2 vessels with moderate amount; 3 vessels with mild amount of plaque
P3	Severe	5–7	301–999	3 vessels with moderate amount; 1 vessel with severe amount of plaque
P4	Extensive	≥ 8	> 1000	Vessels with severe amount of plaque

*SIS* segment involvement score [24]

## CAD-RADS modifiers

**Fig. 3:**
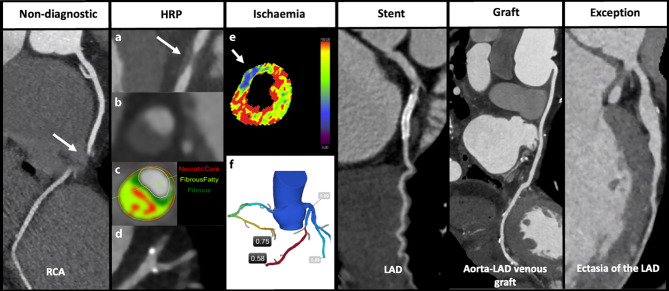
CAD-RADS modifiers: **a** low-attenuation plaque; **b** plaque with positive remodelling and napkin-ring sign; **c** semi-automated assessment of plaque composition; **d** spotty calcifications (< 3 mm); **e** computed tomography perfusion image with perfusion defect (white arrow) in the territory of LAD; **f** fractional-flow reserve (FFR) CT with haemodynamic significant stenosis in the LAD and RCA. *HRP* high-risk plaque*, LAD* left anterior descending, *RCA* right coronary artery

In CAD-RADS 2.0 there are 6 modifiers which can be supplemented to the CAD-RADS category. Potential modifiers should be added to the CAD-RADS category after plaque burden. For all the modifiers see Fig. 3 as well as the teaching website Radiology Assistant. (https://radiologyassistant.nl/cardiovascular/cad-rads/coronary-artery-disease-reporting-and-data-system).

### Modifier non-diagnostic (N)

CAD-RADS N indicates that not all segments with a diameter > 1.5 mm are diagnostic (e.g. segments with motion artifact, calcium blooming, insufficient contrast opacification). In case of minimal or mild stenoses in other diagnostic segments, the study should be graded as CAD-RADS N (in this case modifier N should precede plaque burden, e.g. CAD-RADS N/P1). If the degree of stenosis in a diagnostic segment is more than 50%, the study should be scored as CAD-RADS 3/N (in this case plaque burden will precede modifier N, e.g. CAD-RADS 3/P1/N).

### Modifier ischaemia (I)

This category is added as a modifier to CAD-RADS 2.0. It can be performed on CCTA using fractional flow reserve CT (FFR_CT_) and stress CT perfusion (CTP). These tests can be considered in lesions ranging from 40 to 90%. In case of a positive test, modifier I+ should be added to CAD-RADS category, while a negative test or prior myocardial infraction should be described as I−. When the test is indeterminate and there is questionable or discrepant interpretation, this should be noted as I+/−.

### Modifier high-risk plaque

The following HRP features **(**Fig. 3**) **can be identified [7]:

- Low-attenuation plaque (< 30 HU)
- Positive remodelling (ratio of outer vessel diameter at the site of plaque divided by the average outer diameter of the proximal and distal vessel greater than 1.1)
- Spotty calcification (calcification < 130 HU and < 3 mm)
- Napkin-ring sign (central low-attenuation area adjacent to the lumen and a high ‘ring-like’ attenuation tissue surrounding this central area) [9].

If two or more of these features are identified, the modifier ‘HRP’ should be added to the CAD-RADS category. In the previous CAD-RADS version this was called vulnerable plaque.

### Modifier stent (S) and/or graft (G)

Modifier S (stent) and/or G (graft) can be added based on the presence of either. In case of severe in-stent restenosis, for instance in the proximal left anterior descending artery, CCTA should be graded as CAD-RADS 4A/S. If the stent is patent and there is severe stenosis, for example in the proximal right coronary artery, it should also be graded as CAD-RADS 4A/S. Similar grading should be performed in case of grafts. The bypassed coronary artery should not be graded. The key is to describe the most severe stenosis where further workup might be considered. Plaque burden should be assessed in total for both native coronaries and graft(s).

### Modifier exceptions (E)

This category includes an anomalous origin of the coronary arteries, coronary artery dissection, coronary artery (pseudo) aneurysm, vasculitis, coronary artery fistula, extrinsic coronary artery compression, arterio-venous malformation and other causes [7].

## Strengths and limitations of CAD-RADS

There were several limitations in CAD-RADS 1.0 which were improved in CAD-RADS 2.0. Based on the results of several previous studies [10, 11], plaque burden as an estimate of the overall amount of coronary plaques was added to CAD-RADS 2.0 (previously, diffuse atherosclerosis with mild stenosis fell into the same category as, for instance, a non-calcified plaque with mild stenosis in one coronary artery only), which allows further refinement of patient management, especially optimal medical therapy. A limitation of plaque burden is that it can be determined using various techniques, so one patient can have multiple degrees of plaque burden. In this case it is recommended to list the one with the highest P score [7]. Regarding the location of the most severe stenosis, CAD-RADS 2.0 still omits the segregation of more proximal versus distal stenosis, while proximal plaques demonstrate a higher event rate [12]. Nevertheless, based on emerging evidence [13] CAD-RADS 2.0 has introduced the modifier ischaemia in case of the presence of haemodynamically significant stenosis detected by FFR_CT_ or CTP. Also, in the new CAD-RADS version possible non-atherosclerotic causes of stenosis such as coronary artery dissection are taken into consideration by addition of modifier exceptions. Of note, the modifier HRP (earlier vulnerable plaque) still includes the presence of low-attenuation plaque, positive remodelling, napkin-ring sign as well as spotty calcification. The addition of HRP to the CAD-RADS categories as such is still, however, debatable owning to its fair to moderate inter-rater reliability and limited prognostic value [14]. Finally, CAD-RADS might underestimate progression of coronary sclerosis on follow-up CCTAs compared with segment stenosis and involvement score [15].

## Prognostic value and reliability of CAD-RADS

CAD-RADS allows uniform, standardised reporting of CAD as well as guiding diagnostic and therapeutic decisions in a patient-tailored approach. It facilitates the communication between the interpreting and referring physician and also serves as a valuable educational tool for specialists (especially radiologists and cardiologists) and non-specialists including fellows, residents and researchers.

The prognostic value of CAD-RADS has been investigated in a comprehensive sub-study of the PROMISE trial in patients with stable chest pain [16]. Events encompassed all-cause death, hospitalisation for unstable angina and myocardial infarction [16]. The study demonstrated a clear increase in hazard ratio (HR) with a higher CAD-RADS degree (the unadjusted HR for an event for CAD-RADS 1 increased from 2.66 to 30.80 for CAD-RADS 4B and 5) [16]. In line with this trial, a retrospective study of the SCOT-HEART trial demonstrated that higher CAD-RADS categories are associated with an increased risk of fatal or non-fatal myocardial infarction, with CAD-RADS 4B at the highest risk (HR = 19.14). Interestingly, event rates in CAD-RADS 3 were very similar to those with severe stenosis or occluded coronary [14]. This observation may be associated with the presence of extensive plaque burden in patients with CAD-RADS 3 in the absence of significant stenosis and underlines the previous observation that most myocardial infarctions occur in segments with non-obstructive CAD [17, 18].

Regarding patients presenting with acute chest pain, a comprehensive study in low-to-intermediate risk patients investigated the prognostic value of CAD-RADS for the prediction of major adverse cardiovascular events (MACE) [19]. The study results demonstrated a gradual increase of HR (range 3.2–8.5) from CAD-RADS category 3 to 5. Notably, the presence of HRP was also associated with increased risk (HR = 3.6), independent of the CAD-RADS score. In line with this study, a comprehensive meta-analysis also showed that all HRP features are a strong predictor for MACE [20]. The presence of ≥ 2 HRP features demonstrated the highest risk of MACE (HR = 9.2). Conversely, in the previously mentioned sub-study of SCOT-HEART the presence of HRP was not associated with increased risk [14]. Moreover, the presence of spotty calcification reduced the specificity of this modifier. Despite these discrepancies, according to CAD-RADS 2.0, HRP features should be recognised and included in the standard report since, similar to plaque burden, their presence might require a more aggressive preventive therapy and/or clinical management.

Reliability for CAD-RADS among expert readers was excellent and only marginally better than that of early career readers [21]. Good image quality was associated with stronger reliability compared with moderate image quality [21]. Reliability for the modifiers stent and bypass graft was excellent for both (κ = 1.0); however, the modifier HRP was found to have fair to moderate reliability (κ = 0.40) [21]. In another study inter-rater reliability of the CAD-RADS reporting system was shown to be very good between two readers with 4 and 7 years of experience in CCTA [22]. Reliability was especially high for CAD-RADS 0 (k = 0.965), while CAD-RADS 3 showed the lowest reliability (κ = 0.801) [22]. Reliability for HRP was not evaluated [22].

## Diagnostic value of CAD-RADS

Invasive coronary angiography (ICA) is the reference standard for the diagnosis of anatomic CAD. Furthermore, through measurement of FFR it allows assessment of the haemodynamic significance of a stenosis [23]. Nevertheless, ICA-related complications can occur [24] and costs are considerable [25]. When comparing the CAD-RADS grading system versus clinically positive (e.g. requiring revascularisation/positive FFR/positive stress test/medical therapy for angina in CAD-RADS 4) or clinically negative outcome, it showed excellent classification with no false-positives in the CAD-RADS 0 and 1 groups and only true-positives in CAD-RADS 5 [26]. Accuracy was 98% for CAD-RADS 2, 92% for CAD-RADS 3 and 70% for CAD-RADS 4 [26]. Moreover, the use of FFR_CT_ did not improve radiological assessment of CAD graded by CAD-RADS alone [26]. These results are similar to another study which demonstrated that in case of significant stenosis (CAD-RADS 4 and 5) the accuracy of CAD-RDS was ∼ 99% for both [25].

## Dutch reporting practices and CCTA-based clinical management

Regarding the Dutch reporting practices of CCTA, a previous article found significant differences between hospitals [3]. In 2019 the majority of hospitals performing CCTA used a standard form for reporting. CAD-RADS, however, was used only in 9 (18.8%) of the 48 hospitals. Since the publication of a CAD-RADS overview and practice cases (2019) on the Radiology Assistant online teaching platform a gradual shift was observed towards standardised reporting based on CAD-RADS. In 2023 an agreement between the NVVR, NVVC, the Dutch Society of Clinical Physics (NVKF) and the Dutch Society of Medical Imaging and Radiotherapy (NVMBR) was reached in order to uniform and standardise CCTA reporting based on CAD-RADS 2.0 [27].

Previous studies have demonstrated that higher CAD-RADS categories are associated with increased use of preventative medications [14, 28] as well as a higher rate of revascularisation [14]. In the Netherlands, no data are available regarding CAD-RADS-driven downstream or upstream treatment recommendations. This observation was recognised and is currently being investigated within the framework of the multicentre, randomised CLEAR-CAD trial (https://clearcad.nl). Briefly, based on the CAD-RADS score, included patients with stable chest pain and suspected CAD are categorised into three groups: no-CAD; non-obstructive CAD and obstructive CAD, where the last two groups receive uniform management in the form of optimal medical therapy and only patients with persistent angina undergo ischaemia detection with the option of revascularisation in case of a positive test.

Based on previous estimates [3] roughly 37,000 CCTA examinations are performed annually in the Netherlands. However, the total number of patients, who according to ESC recommendations would require CCTA, is approximately 106,000 patients [3], which means that the number of annual CCTA scans might triple in the near future. This increase warrants not just superior diagnostic assessment but also the use of an insightful reporting system in order to ensure accuracy and effective communication of results and ultimately improved patient care.

## Future perspectives

A potential benefit of CAD-RADS is the use of standardised terminology which might help the development of artificial intelligence (AI) aided reporting of CCTA. Quantitative (e.g.: plaque volume) and qualitative (e.g.: hallmarks of HRP) assessment of coronary plaque composition is emerging and widely used for research; however, variations between different reconstruction algorithms as well as thresholds (e.g. low attenuation plaque 30 HU vs. 60 HU vs. 90 HU) and the time-consuming nature of (semi)-automated measurement methods hamper the implementation of these methods in real-world clinical practice [29]. Recently, AI-driven quantification methods emerged to address this issue. A study by Nurmohamed et al. [30] demonstrated that AI-guided measured atheroma volume of > 15% as well as non-calcified plaque volume of > 7.5% is associated with a 3- and 4‑fold increased MACE risk. Additionally, AI-driven quantification achieved an improved area under the curve compared with CAD-RADS 2.0 (10-year AUC: 0.78; 95% CI: 0.73–0.83; *p* *=* 0.023).

In a previous study, AI demonstrated an excellent performance regarding the accuracy, sensitivity, specificity, positive predictive value and negative predictive of > 70% stenosis (99.7%, 90.9%, 99.8%, 93.3%, 99.9%, respectively) and slightly lower values in case of > 50% stenosis (94.8%, 80.0%, 97.0, 80.0%, 97.0%, respectively) [31]. The overall agreement between experts and AI within one CAD-RADS category was 78.0%, where 98.3% agreed within one category. HRP was identified in 21.1% of patients using AI and in 13.4% of patients by consensus readers with fair reliability (κ = 0.372).

A holistic overview of AI-driven processes in CCTA nicely summarises the available, albeit in clinical workflow limited, technical principles (such as machine learning and radiomics), through which additional aspects of CCTA (such as peri-coronary adipose tissue, epicardial adipose tissue) might be integrated in future CAD-RADS categories [32]. Another possible game-changer regarding CAD-RADS based clinical decisions is the development of photon-counting detector CT (PCD-CT) technology. In a retrospective study, ultra-high-resolution reconstruction led to reclassification of 62 of 114 (54.4%) patients to lower a CAD-RADS category than that assigned using standard resolution [33]. The use of spectral PCD-CT allows sharper and more confined visualisation of calcifications without blooming, which might be especially relevant in patients with excessive plaque burden. Therefore, it is expected that more patients with a higher pre-test probability will be referred for CCTA.

## Conclusion

CAD-RADS enables concise and insightful reporting of CAD. The systematic, standardised approach of reporting CCTA results not only improves communication with referring clinicians but also guides patients’ therapeutic management. The second iteration of CAD-RADS (2.0) addresses the presence and extent of plaque burden. In addition, modifier I (ischaemia) allows the incorporation of other CTA-derived techniques including FFR_CT_ and CTP while modifier E (exception) makes it possible to denote potential coronary abnormalities. It is also anticipated that the use of AI as well as state-of-the art imaging methods such as ultra-high resolution and spectral CCTA will allow better visualisation as well as integration of previously undetectable hallmarks of CAD in future versions of CAD-RADS. The use of CAD-RADS is especially important in the Netherlands where the number of CCTA examinations performed annually is expected to triple in the upcoming years.
